# The influence of cultural attitudes to nut exposure on reported nut allergy: A pilot cross sectional study

**DOI:** 10.1371/journal.pone.0234846

**Published:** 2020-06-19

**Authors:** Lilahom Ben Kayale, Jonathan Ling, Emily Henderson, Noel Carter

**Affiliations:** 1 Faculty of Health Sciences and Wellbeing, University of Sunderland, Sunderland, United Kingdom; 2 Institute of Health and Society, Newcastle University, Newcastle upon Tyne, United Kingdom; Texas A&M University College Station, UNITED STATES

## Abstract

Food allergies in children have become a common management and diagnostic concern and have a significant influence on general health-related quality of life. We investigated the prevalence of reported nut allergy between populations with different cultural attitudes to nuts during pregnancy and infancy. We conducted a survey to investigate the relationship between cultural differences in the consumption of nuts during pregnancy, breastfeeding and exposure to nuts in early childhood against the reported prevalence of nut allergy between three populations: Libyan, UK Libyan and a general UK population. The survey was administered to a representative sample of UK and Libyan parents with children aged between 3 and 16 years who were asked to report prevalence of nut allergy and to describe the factors that might affect this such as cultural behaviours and diet. A total of 1,123 parents responded. Nut allergy was defined as an allergic reaction that required medical treatment. The reported rates of nut allergy showed a significant difference in nut allergy between the Libyan populations and the general UK population with an increased odds ratio of nut allergy of ~10 when comparing the Native Libyan population to the UK population. The UK Libyan population reported the same low rate of allergic reactions as the Libyan population which were both significantly lower than the UK population (p < .0001). The Libyan populations showed significant differences from the UK population in exposure to nuts during pregnancy, breastfeeding and early infancy. The development of peanut and almond allergy through tolerance induction could be prevented by frequent and early ingestion of a moderate quantity of nuts during infancy and by maternal ingestion during pregnancy or lactation.

## Introduction

The prevalence of food allergy within populations is rising and is a growing public health issue [1].Food allergy in children has become a common management and diagnostic concern for the general paediatrician. Such allergies among children have a significant influence on general health-related quality of life, an emotional impact and can limit social activities [2].

Generally, the common allergens responsible for severe reaction in infancy and early childhood are egg, milk, peanut, wheat, and soya. Typical allergens of older children and adults are peanut, tree nuts, and seafood [3]. Currently, the diagnosis and management of food allergy is solely through the avoidance of allergens and the treatment of symptoms [4]

Recent reviews of epidemiological risk factors for food allergy have shown several factors that influence the prevalence of food allergy [3]. The notion that the risk of food allergy may be caused by delaying exposure to allergenic foods in infancy and early childhood is supported by numerous epidemiological studies, and in particular that delaying exposure to allergenic foods is a risk factor for the development of allergies[5,6]. Nevertheless, the consequences of intrauterine exposures, and allergenic food exposure by maternal consumption during pregnancy and lactation remain unclear [7–9].

In the UK, healthcare providers and government experts have long urged expectant and pregnant mothers to avoid peanuts and their by-products to reduce the potential for development of nut allergies in their babies (Committee on Toxicology (COT), 1998). Similar advice was given in the United States Of America (USA), whereby children with a family history of allergies who are at a high risk of atopy should not consume peanuts until after three years of age, although this advice has recently changed to state that peanuts can be introduced when age appropriate and in accordance with family preferences and cultural practices to children who have no eczema or food allergies [10], although the evidence for this was rated in the guidance as being of low quality. Despite such guidance, in both the UK and the USA there is still widespread confusion amongst parents and practitioners over peanut consumption in young children. Despite the recommendation to avoid peanuts, tree nuts, and other food allergens during pregnancy, lactation, and infancy in Australia, the UK, and the USA, the prevalence of peanut and tree nut allergies–along with food allergies more generally—are rising in these countries[11]. An estimate based on UK and USA data is that 1.8 to 2% of children has an allergy to peanuts with0.5% of children having an allergy to tree nuts [12,13].

The failure of avoidance recommendations to reduce the prevalence of allergies may have three potential explanations. First, other routes allow sensitisation to occur and not only through oral exposure but also through cutaneous exposure; second, tolerance induction may need early oral exposure; third, food allergies may develop irrespective of being exposed to the allergen [14].

Nutrition is one environmental factor that can play a major role in the development of asthma and allergies [15]. Age of food introduction, maternal and infant diets, as well as various cultural dietary habits and other dietary patterns result in a different prevalence of food allergy between populations [16].

This work was initiated by contrasting clinical observations by the lead author in her roles as a physician in the UK and Libya. She observed anecdotally that the number of diagnosed mild and severe cases of peanut and almond allergic reactions were less frequent than cases seen in the UK. However, international comparative studies of this type are rare in the Arab world.

In Libya, the traditional diet is characterized by various components of plant foods such as fruits, vegetables, cereals (mainly wholegrain) and different kinds of nuts [17]. As part of this, Libyan children tend to consume high quantities of almonds and peanuts. Early, regular, and frequent consumption may have a protective role against allergic symptoms. Most Libyan children have a moderate to high level of adherence to the dietary pattern of this traditional diet which may explain the low prevalence of nut allergy.

Furthermore, studies have shown that nut sensitization is likely to be a complex mixture of cultural attitudes to exposure and the development of oral tolerance by exposure. A recent study in Australia [18] showed cultural, ethnic and class differences in allergy rates using methodologies similar to this work. The STOP II trail in the UK [19] demonstrated that children that were already sensitized could be successfully desensitised by exposure to nut allergens. Other work has shown that infants sensitive to allergens could be desensitized by exposure and conversely children who were not sensitive to allergens were more likely to develop sensitization if they were not exposed [20].

However, the differing prevalence of nut allergies in the UK compared with those in Libya might be due to genetic differences rather than the differences in cultural dietary patterns, environmental factors, and the age of exposure to food allergens in the two countries. A comparison of Libyan children living in Libya, with Libyan children who live in the UK, who have a similar cultural and genetic background but a different environmental background, and UK children who share similar environmental factors but different cultural and genetic backgrounds to the Libyan children may provide helpful data to clarify this difference. The aim of this study was to investigate if there was a difference in nut allergy between different populations that have a culturally different attitude to the introduction of tree nuts into the diet in early influence.

## Method

### Design

A pilot cross-sectional study was used to describe the prevalence of nut allergy in Libyan and in UK children, to explore influences of culture/diet, and to describe the prevalence in an immunological context. As the population of this study was stratified on more than one variable, opportunity samples were drawn from three groups [21]. In this case, the groups were: British, Libyans living in the UK (UK Libyans) and Libyans living in Tripoli, Libya.

### Participants

A total of 3,300 questionnaires (1,000 questionnaires to primary and secondary schools in Tripoli, Libya; 300 questionnaires to Libyan schools in the UK; and 2,000 questionnaires to British schools in the North East of the UK) were distributed.

The questionnaires were distributed to nine schools in the North East region of UK with a response rate of 13.2%. An Arabic version of the questionnaire was distributed to seven schools in Libya with a response rate of 65.5% and children in two Libyan schools in the UK with a response rate 68%. In total there were 1,123 completed questionnaires returned, giving an overall response rate of 34%. The number of respondents living in Libya was 655 (58.3% of the sample), there were 204 (18.2%) Libyans living in the UK,and 264 (23.5%) of the participants were UK natives. The majority of participants (83.1%; 933) lived in urban areas while 16.9% (190) lived in rural areas. The reported data were based on boys and girls aged between 3 and 16 years with a mean age of 7.7 years at the time of survey.

### Procedure

The questionnaire was given to headteachers to distribute to pupils who were asked to give it to their parents to complete. The questionnaire was in English for both UK-based groups and in Arabic for the Libyan sample.

The questionnaire consisted of a total of 33 questions that covered genetic factors, immunological reactions, and cultural behaviours. The respondents were asked to complete information regarding age, sex, place of residence, cultural background, family allergic diseases, family food allergy and mothers’ nut consumption and avoidance during pregnancy and lactation and the duration of breastfeeding. Respondents were also asked to report hypersensitivity to milk, eggs, sesame, peanut, and tree nuts in their children. They were also asked to report the age of the child when tree nuts and peanuts were first introduced, if or when any symptoms of allergy first occurred, the number of nuts given to the child, the reason for advising the child to avoid consuming nuts, and the type of food containing nuts given to the child. Finally, respondents were asked about the nature and timing of symptoms after exposure to almonds and peanuts, type and place of treatment received and its effectiveness, and predisposing factors. The study was piloted to test the viability and to refine the research tool. The Arabic version was back-translated into English to ensure good agreement between the content of the items on both versions of the questionnaires.

### Ethics

Ethical approval for this project was granted by the University of Sunderland Research Ethics Committee, as well as the Ethics Committee of Tripoli Children’s Hospital. The questionnaires were distributed to parents through a number of schools in Libya and the UK through each school’s head teacher who provided written consent for us to distribute the questionnaire. The questionnaires were distributed to pupils for them to take home to their parents with a participant information sheet and a consent form that was signed by the parent and then returned with the questionnaire. No questionnaires were returned without a written consent form.

### Data analysis

Responses were coded and the data was placed into a database using Statistical Package for the Social Sciences (SPSS) version 21. Descriptive analysis was used to calculate the proportion of each group of respondents with each statement in the questionnaire. Subsequent analysis was performed using different tools. A Fisher’s exact test was performed using the statistical programming language R v3.5.2 [22] running in RStudio [23] and post-hoc testing of the chi-squared results was performed using the package R commander [24] to generate adjusted p values using a Bonferroni correction with alpha set at 0.05. Odds ratios and relative risk were also calculated using online tools [25] and as three pairwise comparisons were performed on the same data, the initial setting of an alpha of 0.05 for significance was adjusted using a Bonferroni correction set at 0.166. A copy of the questionnaire and the SPSS data file can be accessed through the open access data archive, DANS (https://doi.org/10.17026/dans-zkg-rt3h)

## Results

### Consumption of nuts during pregnancy

Overall, almost 95% of mothers reported eating nuts or foods containing nuts during pregnancy (see Table 1). Almost all Libyans and UK Libyans reported consuming nuts or foods containing nuts during pregnancy, as did the majority of UK natives. Of the 58 participants who did not eat any nuts or foods containing nuts, 50 were UK natives. Statistical analysis compared groups based upon whether nuts (or food containing nuts) were avoided during pregnancy. In comparison to both groups of Libyans, the UK natives had a lower than expected intake of nuts or food containing nuts during pregnancy; Libyan versus UK (adjusted p = 2.55e-21), UK Libyan versus UK (adjusted p = 3.72e-12), Libyan versus UK Libyan (adjusted p = 1.0).

**Table 1:** Cultural population and nut consumption during pregnancy.

		Cultural population		
	Counts	Libyan^ac^	UK Libyan^ab^	UK^bc^
Consumed nuts during pregnancy	N	648	203	214
	%	98.93	99.50	81.06
Avoided nuts during pregnancy	N	7	1	50
	%	1.07	0.50	18.94

Statistical analysis was performed using a Fisher’s exact test with Bonferroni correction and the adjusted p values were as follows (a) NS (b) p<0.001 (c) p<0.001.

### Breastfeeding

Most participants (90.7%; 1018) consumed nuts or foods containing nuts at least once during breastfeeding (see Table 2). Almost all Libyans reported eating nuts or foods containing nuts at least once during breastfeeding, with the majority of UK natives also reporting consumption. Of the 105 participants who did not eat any nuts or foods containing nuts, 80 were UK natives. A higher percentage of Libyans ate nuts or food containing nuts during breastfeeding compared to UK natives; Libyan versus UK (adjusted p = 9.33e-32), UK Libyan versus UK (adjusted p = 1.87e-13), Libyan versus UK Libyan (adjusted p = 0.462)

**Table 2:** Cultural population and nut consumption during breastfeeding.

		Cultural population		
	Counts	Libyan^a,c^	UK Libyan^a,b^	UK^b,c^
Consumed nuts during breastfeeding	N	639	195	184
	%	97.56	95.59	69.70
Avoided nuts during breastfeeding	N	16	9	80
	%	2.44	4.41	30.30

Statistical analysis was performed using a Fisher’s exact test with Bonferroni correction and the adjusted p values were as follows (a) p<0.01 (b) p<0.001 (c) p<0.001.

### Nut exposure during infancy

Of the 1,123 participants, 30.3% (340) were exposed to peanuts before 12 months rising to 69.7% (783) being exposed before 24 months of age (see Table 3). There was a statistically significant difference between groups and peanut exposure during infancy at 12 months, Libyan versus UK (adjusted p = 4.62e-09), UK Libyan versus UK (adjusted p = 2.36e-13), Libyan versus UK Libyan (adjusted p = 5.73e-03). At 24 months Libyan versus UK (adjusted p = 9.75e-08), UK Libyan versus UK (adjusted p = 3.63e-11), Libyan versus UK Libyan (adjusted p = 3.03e-03). In this case there was a significantly difference in exposure to peanuts between the Libyan and the UK Libyan population, with the UK Libyan population exposing their children to peanuts during infancy at the highest rate (Table 3).

**Table 3:** Cultural population and exposure to peanuts during infancy at 12 months and 24 months (cumulative).

		Cultural population		
	Counts	Libyan^a,c^	UK Libyan^a,b^	UK^b,c^
Exposed to peanuts during first 12 months of infancy	N	213	91	36
	%	32.52	44.60	13.64
Not exposed to peanuts during the first 12 months of infancy	N	442	113	228
	%	67.48	55.40	84.36
	Counts	Libyan^d,f^	UK Libyan^d,e^	UK^e,f^
Exposed to peanuts during first 24 months of infancy	N	449	164	129
	%	68.55	80.39	48.86
Not exposed to peanuts during the first 24 months of infancy	N	206	40	135
	%	31.45	19.61	51.14

Statistical analysis was performed using a Fisher’s exact test with Bonferroni correction and the adjusted p values were as follows (a) p<0.01 (b) p<0.001 (c) p<0.001 (d) p<0.01 (e) p<0.001 (f) p<0.001

Of the 1,123 participants, 55.1% (619) were exposed to almonds before 12 months rising to 75.8% (851) being exposed before 24 months of age (see Table 4). There were statistically significant differences between groups and almond exposure during infancy; at 12 months, Libyan versus UK (adjusted p = 4.44e-147), UK Libyan versus UK (adjusted p = 1.32e-32), Libyan versus UK Libyan (adjusted p = 1.0). At 24 months Libyan versus UK (adjusted p = 3.75e-33), UK Libyan versus UK (adjusted p = 1.88e-19 Libyan versus UK Libyan (adjusted p = 1.0). In this case the two Libyan populations did not have significantly different exposures to almonds.

**Table 4:** Cultural population and exposure to almonds during infancy at 12 months and 24 months (cumulative).

		Cultural population		
	Counts	Libyan^a,c^	UK Libyan^a,b^	UK^b,c^
Exposed to almonds during first 12 months of infancy	N	437	141	41
	%	66.72	69.12	15.53
Not exposed to almonds during the first 12 months of infancy	N	218	63	223
	%	33.28	30.88	84.47
	Counts	Libyan^d,f^	UK Libyan^d,e^	UK^e,f^
Exposed to almonds during first 24 months of infancy	N	558	174	119
	%	85.19	85.29	45.08
Not exposed to almonds during the first 24 months of infancy	N	97	30	145
	%	14.81	14.71	54.92

Statistical analysis was performed using a Fisher’s exact test with Bonferroni correction and the adjusted p values were as follows (a) NS (b) p<0.001 (c) p<0.001 (d) NS (e) p<0.001 (f) p<0.001

### Nut allergy

Nut allergy was defined by those respondents who stated that exposure to nuts always required medication. The total prevalence of peanut allergy in the study sample was 3.1% (35 per 1,123 cases). By cultural background the prevalence was 0.4% (3 per 655 cases) for Libyans, 0.4% (1 per 204 cases) for UK Libyans, and 4.5% (2 per 264 cases) for UK participants (Table 5). Statistical analysis showed that there was a significant difference between the Libyan and UK groups for nut allergy; Libyan versus UK (adjusted p = 1.48e-4), UK Libyan versus UK (adjusted p = 0.0259), Libyan versus UK Libyan (adjusted p = 1.0). The odds ratio between the Libyan and UK population was 9.93 (2.7–33.5 95% Cl, z = 3.53, p< 0.004) and between the UK Libyan and UK population was 9.27 (1.2–71.9 95% Cl, z = 2.13, p< 0.033), however, this was not significant after performing a Bonferonni correction. Between the Libyan and UK Libyan population the odds ratio was 1.0706 (0.1108–10.3493 95% Cl, z = 0.059, p = 0.9530) making it effectively identical.

**Table 5:** Cultural population and nut allergy.

		Cultural population		
	Counts	Libyan^a,c^	UK Libyan^a,b^	UK^b,c^
No nut allergy that always required medical intervention	N	652	203	252
	%	99.54	99.50	95.45
Allergy that always required medical intervention	N	3	1	12
	%	0.46	0.50	4.55

Statistical analysis was performed using a Fisher’s exact test with Bonferroni correction and the adjusted p values were as follows (a) NS (b) p<0.05 (c) p<0.001

## Discussion

The hypothesis of this study, that variation in the development of nut allergies in children may be a result of cultural behaviors, was based on anecdotal observations that cultural differences influence whether children ingest nuts in early childhood. Moreover, in populations where nut exposure is actively avoided, there appeared to be more nut allergy cases than in populations that encourage early introduction of nuts.

One of the main findings of this research is that the development of peanut and almond allergy, likely through tolerance induction, appears to be lower where frequent ingestion of a moderate quantity of peanut and almond is observed during early infancy. We found that the most obvious difference in the diet of children across populations occurred in the introduction of almonds and peanuts. Libyan children were introduced to almonds and peanuts earlier in life than UK children; the UK population had significantly less exposure during early infancy. The quantity of peanuts introduced to the UK Libyans and UK participants was similar (unpublished); but the age at which the UK participants introduced peanuts to their children was different: by the age of two only 45% of the UK population had been exposed to nuts compared to ~85% in both Libyan populations. This later introduction is likely to have been influenced by the Department of Health recommendation that children should not be exposed to nuts until three years of age [11]. However, this advice has now been revised and the current NHS advice now is not to expose children to nuts before the age of 6 months and this advice has also been amended in the USA [10]. A similar study in Australia [18] using a similar methodology to this study reported that Asian children born in Australia had a higher incidence of nut allergy than Asian children who had come to Australia through migration.

In previous work [7,16,26,27], dietary patterns were found to influence the prevalence of individual food allergies. These epidemiologic studies demonstrated that the likelihood of an allergy or tolerance developing can be determined by the age of exposure to food allergens. This remains the most convincing explanation, and furthermore, the risk of developing a food allergy may be *increased* by delaying the exposure to allergenic foods in infancy. This research supports the notion that the early introduction of food allergens seems to protect infants from food allergies.

Tolerance can also be potentially induced by the early oral introduction of peanuts and almonds [28–30]. Specifically, the results of our study support the hypothesis that oral tolerance induction by exposure to peanuts and almonds in early infancy (six to 12 months) may reduce allergy. The recent STOP II [19] trial has demonstrated that children who are highly sensitive to peanut allergens could have their sensitivity reduced by repeated exposure to the allergen. Although the precise mechanism for this action is unclear, the study demonstrated that the reduction in sensitization was clinically relevant and offers hope that sensitization can at least be reduced long term. However, a recent study by Du Toit [20] has provided more mechanistic evidence that oral tolerance is a key component in the development of desensitization to nut allergens in infants. In the study, infants were tested for sensitivity and separated into two cohorts who were then subdivided into groups that avoided or were exposed to nuts. In the sensitive cohort, those who were exposed to nuts had reduced sensitization, but conversely in the non-sensitive group avoidance led to an increase in sensitivity.

While genetic differences cannot be discounted for the differences observed in this study, we did not test these. Work focusing on whether siblings also have similar allergies would be an interesting addition to the field, as would comparing parental responses with children’s clinical records.

While a higher percentage of Libyan mothers who live in the UK or in Libya consumed nuts or food containing nuts during pregnancy and breastfeeding than UK participants., we found that maternal nut ingestion during pregnancy and lactation did not appear to increase reports of nut allergy in children. In addition, there was no association between nut allergy and duration of breastfeeding. The suggestion that breastfeeding can have a protective effect if continued for at least 12 months and may prevent the development of a nut allergy was unsupported. There was no association between the reported length of time of breastfeeding and the number of reported cases of nut allergy.

This study shows the prevalence of almond and peanut allergy is lower in Libyan children compared to the percentage in our general UK control population. We found no evidence of freedom from allergy to peanuts and almonds when consumption of these was delayed.

There are some limitations to this study. The study was questionnaire-based with no clinical data collected and therefore relies on the accuracy of the recollections of the respondents. Recall can be inaccurate and biased, and the age range of the children–from 3–16 years–means some parents may have forgotten early life events. Nonetheless, parents who find their children have allergies or sensitivity are more likely to recollect (and possibly exaggerate) the number of nuts consumed, as well as the severity of the symptoms. If this were the case in this study, this would have increased the values for nut consumption in the general UK population which reported a statistically significant lower consumption overall. The response rate overall was 34%. With a response rate of 13.2% from the UK group that may have led to less accurate estimates of prevalence of allergy and was roughly one fifth of the response rate compared to either Libyan populations. This is likely to contribute to some level of bias with parents of children suffering from nut allergies much more likely to respond. However, we feel that the relatively large number of respondents and the significance of the effects noted here indicate that, even if overestimated slightly, that there remains a significant difference between the UK and Libyan populations.

## Conclusion

We found a strong inverse association between almond and peanut consumption during infancy and the reported prevalence of allergies to peanuts and almonds. Differences in ancestry, cultural beliefs, environmental factors, and atopy could be responsible for variations in nut allergy prevalence between Libyan and UK children. Although the UK Libyan population shares cultural and genetic properties with the native Libyans, this population also shared some cultural and environmental factors with the general UK population. However, the data presented here suggest there is little difference in nut allergy prevalence between the two Libyan populations due to the effect of the general environment, and that cultural practices, namely the introduction of nuts in infancy, are the most likely reason for the lower prevalence of nut allergy in these groups when compared to the general UK population.

Our analyses showed a difference in nut allergy prevalence between Libyan and UK children. While we cannot say for certain whether this difference is caused by culture, genetic, social, or other differences between these two population, data from parents supports the notion that that this difference in nut allergy is due to early exposure. However, there may be other reasons for this difference, or an interaction between different variables. Further work is therefore required to determine conclusively whether current infant feeding recommendations on nuts should be changed.
